# Prevalence and its associated factors of diabetic retinopathy among type 1 and type 2 diabetic patients at public hospitals in Eastern Ethiopia, 2023: a hospital-based comparative cross-sectional study

**DOI:** 10.3389/fcdhc.2024.1432551

**Published:** 2024-11-14

**Authors:** Feyisa Shasho Bayisa, Teshome Demis Nimani, Samuel Demissie Darcho

**Affiliations:** School of Public Health, College of Health and Medical Sciences, Haramaya University, Harar, Ethiopia

**Keywords:** diabetes mellitus, diabetic retinopathy, Ethiopia, type 1 DM, type 2 DM

## Abstract

**Introduction:**

Diabetic retinopathy (DR) is a highly prevalent microvascular disease among diabetic patients, resulting in irreversible blindness. However, there is a dearth of evidence on diabetic retinopathy (DR) and its associated factors in eastern Ethiopia. The study aimed to determine the prevalence of diabetic retinopathy (DR) and its associated factors among type 1 and type 2 diabetic patients at public hospitals in eastern Ethiopia.

**Method:**

A hospital-based comparative cross-sectional was conducted among 520 diabetic patients. Epidata software was used for data entry, and STATA version 17 was used for statistical analysis. Multivariate binary logistic regression was computed to identify factors associated with DR. The Hosmer and Lemeshow chi-square test assessed goodness of fit.

**Results:**

The overall prevalence of DR was 43.5%. The prevalence of diabetic retinopathy among type 1 DM was 38.5%, and the prevalence of DR among type 2 DM was 48.5%. Age >60 [AOR = 4.64 95% CI (1.60, 13.51)], being male [AOR = 4.05 95% CI (1.51, 10.97)], and having complications [AOR = 0.01 95% CI (0.003, 0.04)] were significantly associated with DR among type 1 diabetes. Having a family history of DM [AOR = 1.57 95% CI (1.76, 3.24)], poor glycemic status [AOR = 1.91 95% CI (1.56, 2.83)], and having complications [AOR = 11.07 95% CI (4.89, 25.13)] were significantly associated with DR among type 2 diabetes.

**Conclusions:**

In the current study, the prevalence of DR was 43.5%. The prevalence was higher among type 2 diabetes compared to type 1 diabetes. Factors such as poor glycemic control, older age, male sex, a family history of diabetes, and complications related to diabetes were significantly associated with DR. To minimize the impact of diabetics, it requires regular screening programs for diabetic patients, especially those with poor glycemic control and other identified risk factors.

## Introduction

Diabetic retinopathy (DR) is an eye-threatening microvascular consequence of diabetes mellitus (DM), resulting in micro aneurysm, hard exudates, hemorrhage, venous changes, cotton-wool spots, and new vessel formation involved in the peripheral retina, macula, or both (1). It is caused by long-term exposure to metabolic changes associated with DM, which result in damage to the retina’s microvasculature (2). There are two types of diabetic retinopathy (DR): nonproliferative (NPDR) and proliferative (PDR) (3). NPDR refers to the absence or presence of abnormal new blood vessels emanating from the retina. PDR is the more advanced form of DR, in which circulation problems deprive the retina of oxygen. If it is not treated, PDR will result in blindness among 50% of diabetic patients (4). Diabetic retinopathy affects almost all patients with type 1 diabetes (DM1) and more than 60% of people with type 2 diabetes (T2DM) (5).

Globally, approximately 95 million (35.4%) diabetic patients had diabetic retinopathy (DR), of which one-third have vision-threatening DR and 7.6% have macular edema (6). The global annual prevalence of diabetic retinopathy is 2.2%–12.7% (7). Worldwide, the prevalence of blindness is estimated to be 1.5 billion, of which 0.4 million is due to diabetic retinopathy (DR). The prevalence of blindness due to diabetic retinopathy (DR) was increased from 0.2 million to 0.4 million and moderate-severe visual impairment from 1.4 million to 2.6 million from 1990 to 2015 (8). The prevalence of diabetic retinopathy was lowest in South and Central America (13.37%) and highest in Africa (35.90%), North America, and the Caribbean (33.30%) (9).

In Africa, around 30 to 31.6% of diabetic patients have retinopathy, and this condition is becoming more common in sub-Saharan Africa (SSA) countries, placing a significant financial burden on these nations (5, 10). In Ethiopia, the prevalence of diabetic retinopathy was 19.48%, with the Oromia region having the highest incidence (24.8%) and the Amhara region having the lowest prevalence (19.99%) (10). Additionally, the previous study showed that the prevalence of diabetic retinopathy in northwest Amhara was 34.6% and 34.1% (11, 12), and it was 41.3% in Jimma (13).

Sociodemographic characteristics such as age, sex, body mass index (BMI), hypertension, poor glycemic control, type 2 diabetes mellitus, high blood pressure, cholesterol level, and duration since diabetes diagnosis are the most common risk factors associated with diabetic retinopathy (5, 10–15).

However, there is a dearth of evidence on diabetic retinopathy (DR) and its associated factors in eastern Ethiopia. There is no prior cross-sectional comparative study conducted in the current study area. Therefore, the current study aimed to determine the prevalence of diabetic retinopathy and its associated factors among type 1 and type 2 diabetic patients in Harar town, eastern Ethiopia.

## Methods and materials

### Study design, period, and setting

A hospital-based comparative cross-sectional study was conducted among diabetic patients by reviewing their medical records. Data were reviewed from November to January 2023 at Jugal Specialized Hospital in Harar. Harar town is the administrative hub of the Harari region, eastern Ethiopia, which is located 526 kilometers from Addis Ababa, the capital city of Ethiopia. According to the 2007 Census, 183,415 people were living in Harari Regional State, and 91,099 of the people were women. There are six hospitals in the city; two of them are government hospitals, two are private, and two are military hospitals. The research was carried out at Jugal Specialized Hospital, which is one of the two public hospitals in Harar city.

### Population

#### Source population

All adult patients with type 1 and type 2 DM are attending the Endocrinology and Diabetes Clinic and the Medical and Surgical Departments of Jugal Hospital.

#### Study population

All adult patients with type 1 or type 2 diabetes attending the Endocrinology and Diabetes Clinic and the Medical and Surgical Departments of Jugal Hospital during the study period who met the inclusion criteria.

### Inclusion and exclusion criteria

#### Inclusion criteria

All consenting adult patients with type 1 and type 2 diabetes who attended the hospital follow-up clinic were included.

#### Exclusion criteria

Diabetic patients under the age of 18 and patients with incomplete information cards were excluded.

### Sample size determination

The sample size for the study was determined by doubling the proportion of the population, assuming a prevalence of DR was 46.2% for type 1DM and 41.7% for type 2DM (16). A power of 80% was used to determine the reported difference between the DR among type 1DM and DR among type 2DM populations, with a confidence level of 95% and a non-response rate of 10%. Consequently, the calculated sample size was 260 for patients with type 1DM and 260 for patients with type 2DM.


$$(17)\text{n}=\frac{{\left(\frac{z\alpha }{2}+z1-\beta \right)}^{2}p1(1-p1)+p2(1-p2)}{{\left(p1-p2\right)}^{2}}$$

### Sampling technique

First, cards of diabetic patients were grouped into type 1 DM and type 2 DM in order to assess the prevalence of diabetic retinopathy (DR) in the two types of diabetic mellitus. Then, by using simple random sampling technique, cards of diabetic patients were selected from each group.

### Data collection instruments

The cohort data from the medical records were obtained using a standardized checklist, which is adapted from a previous study. The checklist was pretested at Hiwot Fana Hospital, which is found outside of the study area.

### Data collection procedure

A health information worker removed the patient folder from the card room after obtaining the medical records of multiple diabetes patients from the chronic care follow-up clinic. Data extractors received training in data extraction. The principal investigators assisted three Bachelor of Science (BSc) nurses in their examination of the records.

### Study variables

#### Dependent variable

Diabetic retinopathy among type 1DM and type 2DM.

#### Independent variables

Sociodemographic information, patient age, sex, residential location, marital status, educational status, history of social drug use, clinical variables including type of DM, duration of DM, history of complications of DM (hypertension, dyslipidemia, nephropathy, neuropathy, PVD), medication adherence, family history of diabetes, and glycemic control.

### Operational definition

#### Diabetic retinopathy

An ophthalmologist verified the diagnosis of DR status by looking at the patient’s visual acuity test results, slit lamp examination results, and direct ophthalmoscope examination results (15).

#### Hypertension

Was defined according to the JNC-8 criteria as a positive history of hypertension, the use of antihypertensive drugs, or blood pressure equal to or greater than 140/90 mmHg measured using a standard procedure (17).

#### Dyslipidemia

Was defined using the adult treatment panel III (ATP III) guidelines when one or all of the following was found: 12 Total cholesterol > 200 mg/dl (5.2 mmol/l), LDL > 100 mg/dl (2.6 mmol/l), Triglycerides > 150 mg/dl (1.7 mmol/l), and HDL < 40 mg/dl (1.03 mmol/L) in men or < 50 mg/dl (1.30 mmol/L) in women (18).

#### Glycemic control

Was defined based on the ADA 2006 clinical recommendation for standards of medical care in diabetes. Good glycemic control was defined as HbAlc < 7%, FPG = 4.4–7.2 mmol and 2-hour postprandial glucose ≤10 mmol/L (18).

#### Type 1 DM

Diabetes diagnosed before 30 years of age and whose initial treatment is Insulin (19).

#### Type 2 DM

Diagnosis of diabetes after 30 years of age and whose initial treatment does not include Insulin (19).

#### Medication adherence

The study participants who answered below the median value of the 7-point questions on treatment adherence were considered to have poor adherence to DM medication. However, the study participants who scored above or equal to the median were classified as having good adherence to DM medication (20, 21).

### Statistical analysis

Data were entered into the Epidata version 4.6 software after they had been extracted to be cleaned, coded, categorized, combined, and examined for completeness. STATA version 17 was used to analyze the data, which were then summarized into variables using frequency, graphs, percentages, mean, and standard deviation. An examination of bivariate binary logistic regression with a cut-off point P value <0.05 was used to identify possible candidate predictors for the entire model. The study used multivariate logistic regression to determine the independent impact of factors on diabetic retinopathy. The Hosmer and Lemeshow chi-square test was used to determine the overall goodness of fit. The adjusted odds ratio was used to summarize the relationship between the predictors and the odds of DR, and the statistical significance was tested with a p-value < 0.05. As a result of the goodness-of-fit test assessment, the Hosmer Lemeshow for type 1 DM and type 2 DM were (p = 0.63) and (p = 0.87), respectively.

### Ethical consideration

An ethical clearance was obtained from the Institutional Health Review Committee at Haramaya University (Ref. No. IHRERC/323/2023). Access to patient medical records required obtaining waiver letters from the Jugal Hospital medical director and permission from the Harari City Administration Health Bureau. There was no requirement for a consent form from subjects directly because the study used secondary data. The study used the fact that all procedures were followed under applicable laws and guidelines.

## Results

### Sociodemographic and clinical characteristics of patients

Around one-third (36.28%) of the study participants with type 1DM were under 60 years of age, and about 35.84% of the study participants with type 2 DM were aged 41 to 60 years. Less than one-third (24.81%) of the study participants were women, and more than two-fifth (23.88%) were urban dwellers. More than half (66.13%) of the study participants had a family history of DM, and around 14.42% of patients with complications of retinopathy adhered to their medication. Close to one-third (30.97%) of patients with type 1 DM had poor glycemic control, and around two-fifths (21.24%) of patients with type 2 DM had poor glycemic control. The majority of patients with type 2 DM (47.79%) had comorbidities. Close to half (49.12%) of patients with type 2 DM had various complications, and around 42.92% of patients with type 1 DM had various complications. (**Table 1**).

**Table 1:** Descriptive statistics of patients with diabetes mellitus with diabetic retinopathy in the Jugal Specialized Hospital in Harar, 2023 (n = 520).

Variables	Categories	Diabetic Retinopathy			
		Yes		No	
		Type 1DM	Type 2DM	Type 1DM	Type 2DM
Age	18-40 41-60 >60	10 (4.42%) 8 (3.54%) 82 (36.28%)	17 (7.52%) 81 (35.84%) 28 (12.39%)	49 (16.67%) 34 (11.56%) 77 (26.19%)	17 (5.78%) 91 (30.95%) 26 (8.84%)
Gender	Male Female	40 (17.70%) 60 (26.55%)	57 (25.22%) 69 (30.53%)	91 (30.95%) 69 (23.47%)	70 (23.81%) 64 (21.77%)
Residence	Urban Rural	88 (38.94%) 12 (5.31%)	32 (14.16%) 94 (41.59%)	141 (47.96%) 19 (6.46%)	21 (7.14%) 119 (38.44%)
Marital status	Single Married Divorced/widowed	17 (7.52%) 71 (31.42%) 12 (5.31%)	15 (6.64%) 94 (14.59%) 17 (7.52%)	44 (14.97%) 100 (34.04%) 16 (5.44%)	16 (5.44%) 101 (34.35%) 17 (5.78%)
Educational status	Illiterate Primary school Secondary school College and above	7 (3.10%) 45 (19.91%) 16 (7.08%) 32 (14.16%)	30 (13.27%) 19 (8.41%) 9 (3.98%) 68 (30.09%)	11 (3.74%) 66 (22.45%) 30 (10.20%) 53 (18.03%)	40 (13.61%) 24 (8.16%) 17 (5.78%) 53 (18.03%)
Occupational status	Housewife Go ‘vent employee Farmer Retirement Private employee Others	22 (9.73%) 29 (12.83%) 12 (5.31%) 19 (8.41%) 11 (4.87%) 7 (3.10%)	22 (9.73%) 26 (11.50%) 12 (5.31%) 33 (14.60%) 23 (10.18%) 10 (4.42%)	26 (8.84%) 35 (11.90%) 28 (9.52%) 37 (12.59%) 24 (8.16%) 10 (3.40%)	22 (7.48%) 26 (8.84%) 12 (4.08%) 31 (10.54%) 32 (10.88%) 11 (3.74%)
Social drug use	Alcohol Khat None	13 (5.75%) 5 (2.21%) 82 (36.28%)	15 (6.64%) 5 (2.21%) 106 (46.90%)	25 (8.50%) 6 (2.04%) 129 (43.88%)	11 (3.74%) 10 (3.40%) 113 (38.44%)
Family history DM	Yes No	62 (27.43%) 38 (16.81%)	87 (38.70%) 39 (17.26%)	96 (32.65%) 64 (21.77%)	51 (17.35%) 83 (28.23%)
Drug adherence	Non-adhered adhered	72 (31.86%) 28 (12.39%)	79 (34.96%) 47 (20.80%)	85 (28.91%) 75 (25.51%)	86 (29.25%) 48 (16.33%)
Duration of diabetes	1-5 6-10 >10	31 (13.72%) 18 (7.96%) 51 (22.57%)	43 (19.03%) 38 (16.81%) 45 (19.91%)	54 (18.37%) 29 (9.86%) 77 (26.19%)	62 (21.09%) 37 (12.59%) 35 (11.90%)
Glycemic level	Poor Good	70 (30.97%) 30 (13.27%)	48 (21.24%) 78 (34.51%)	103 (35.03%) 57 (19.39%)	73 (24.83%) 61 (20.75%)
Comorbidity	Yes No	46 (20.35%) 54 (23.89%)	108 (47.79%) 18 (7.96%)	38 (12.93%) 122 (41.50%)	50 (17.01%) 84 (28.57%)
Baseline FBS	Mean ± SD	130.6 ± 26.8	160.5 ± 73.8	136.9 ± 31.4	154.5 ± 52.4
Complications	Yes No	97 (42.92%) 3 (1.33%)	111 (49.12%) 15 (6.64%)	47 (15.99%) 113 (38.44%)	39 (13.27%) 95 (32.31%)

### Prevalence of diabetic retinopathy

**Figure 1:**
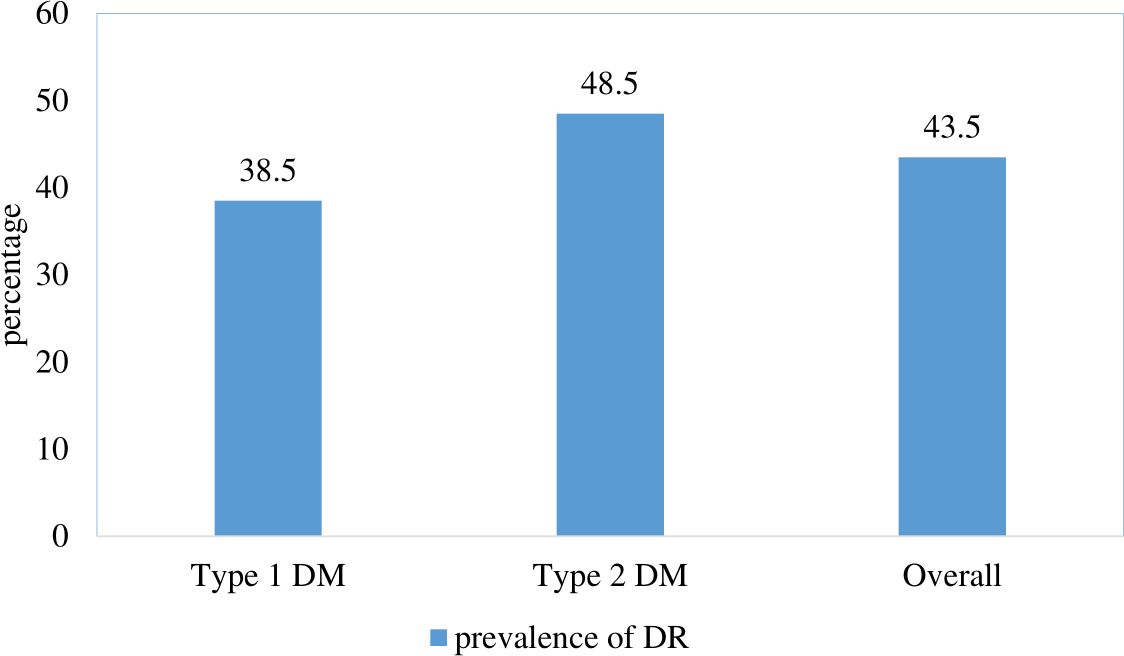
Prevalence of diabetic retinopathy among patients with diabetes mellitus at Jugal Specialized Hospital, Harar, Ethiopia, 2023.

The overall prevalence of complications of diabetic retinopathy in this study was 226 or 43.5% (95% CI: 39.2%, 47.8%). The prevalence of retinopathy among type 1DM was 38.5% (95% CI: 32.5%, 44.7%) and among type 2DM 48.5% (95% CI: 42.2%, 54.7%). (**Figure 1**).

### Factors associated with diabetic retinopathy among patients with type 1 DM

In the bivariate logistic regression, age, sex, marital status, occupational status, adherence to medications, average FBS, complication, and comorbidity were eligible for multivariate analysis. In the multivariate logistic regression analysis, age, sex, and complications had a significant influence on the development of diabetic retinopathy among patients with type 1 DM. The odds of developing diabetic retinopathy were 4.64 [AOR = 4.64 (95% CI (1.60-13.51)] times higher among patients aged > 60 years compared to patients aged 18-40 years. The odds of developing diabetic retinopathy were 4.05 [AOR = 4.05, 95% CI (1.51-10.97)] times higher among male patients compared to female patients. The odds of developing diabetic retinopathy were 0.01 [AOR = 0.01, (95% CI (0.003, 0.04)] times less likely among patients who had no complications compared to patients who had complication. (**Table 2**).

**Table 2:** Baseline factors associated with diabetic retinopathy among patients with type 1 diabetes mellitus at Jugal Specialized Hospital, Harar, Ethiopia, 2023.

Variable	Categories	DR status		COR 95% CI	AOR 95% CI
		No	Yes		
Age	18-40 41-60 >60	49 34 77	10 8 82	1 1.15 (0.41, 3.22) 5.22 (2.47, 11.02)	1 1.48 (0.35, 6.21) 4.64 (1.60, 13.51)**
gender	Female Male	91 69	40 60	1 1.98 (1.19, 3.29)	1 4.05 (1.51, 10.97)**
Marital status	Single Married Divorce	44 100 16	17 71 12	1 1.84 (0.97, 3.47) 1.94 (0.76, 4.94)	1 2.42 (0.92, 6.41) 2.36 (0.54, 10.29)
Occupational status	House wife Government-organization Farmer Retirement Private- organization others	26 35 28 37 24 10	22 29 12 19 11 7	1 0.98 (0.46, 2.08) 0.51 (0.21, 1.22) 0.61 (0.27, 1.34) 0.54 (0.22, 1.35) 0.83 (0.27, 2.54)	1 1.008 (0.34, 3.03) 3.04 (0.59, 15.67) 1.44 (0.43, 4.80) 1.65 (0.38, 7.14) 1.14 (0.23, 5.73)
Drug adherence	Adhered Non-adhered	75 85	28 72	1 2.27 (1.33, 3.88)	1 0.90 (0.38, 2.13)
Average FBS				0.99 (0.98, 1.01)	1.00 (0.99, 1.02)
Complication	Yes No	47 113	97 3	1 0.01 (0.004, 0.04)	1 0.01 (0.003, 0.04)**
Comorbidity	No Yes	122 38	54 46	1 2.73 (1.60, 4.67)	1 0.86 (0.38, 1.96)

** statistically significant.

### Factor associated with diabetic retinopathy among patients with type 2 DM

In the bivariate logistic regression, gender, residence, social drug use, family history of DM, duration of DM, glycemic status, complications, and comorbidity were candidates for multivariate regression analysis. In multivariate logistic regression analysis, the history of DM, glycemic level, and complications of DM were statistically significantly associated with the development of diabetic retinopathy among patients with type 2 DM. The odds of developing diabetic retinopathy were 1.57 [AOR = 1.57, 95% CI (1.76, 3.24)] times higher among patients with a family history of DM compared to patients without a family history of DM. The odds of developing diabetic retinopathy were 1.91 [AOR = 1.91, 95% CI (1.56, 2.83)] times higher among patients with poor glycemic control compared to patients with good glycemic control. The odds of developing diabetic retinopathy were 11.07 (AOR = 11.07, 95% CI (4.89, 25.13) times higher among patients with complications compared to patients without complications. (**Table 3**).

**Table 3:** Baseline factors associated with diabetic retinopathy among type 2 diabetes mellitus in Jugal Hospital, Harar, Ethiopia, 2023.

Variable	Categories	DR status		COR 95% CI	AOR 95% CI
		No	Yes		
gender	Male Female	70 64	57 69	1 1.32 (0.81, 2.16)	1 1.28 (0.62, 2.65)
Residence	Urban Rural	21 113	32 94	1 0.55 (0.29, 1.01)	1 0.98 (0.44, 2.19)
Social drug use	Alcohol use Chat use None	11 10 113	15 5 106	1 0.37 0.69 (0.31, 1.56)	1 0.30 (0.05, 1.60) 0.66 (0.22, 1.99)
Family history DM	No Yes	83 51	39 87	1 3.63 (2.17, 6.07)	1 1.57 (1.76, 3.24)**
Duration of DM	1-5 6-10 >10	62 37 35	43 38 45	1 1.48 (0.82, 2.69) 1.85 (1.03, 3.34)	1 1.38 (0.61, 3.12) 1.08 (0.48, 2.40)
Glycemic status	Good Poor	73 61	48 78	1 1.94 (1.18, 3.19)	1 1.91 (1.56, 2.83)**
Complication	Yes No	39 95	111 15	18.03 (9.36, 34.72) 1	11.07 (4.89, 25.13) ** 1
Comorbidity	No Yes	84 50	18 108	1 10.08 (5.48, 18.54)	1 2.30 (0.92, 5.75)

** statistically significant.

## Discussion

The study aimed to determine the prevalence of diabetic retinopathy (DR) and its associated factors among type 1 and type 2 diabetic patients at public hospitals in eastern Ethiopia.

In the current study, the overall prevalence of diabetic retinopathy (DR) was 43.5% (95% CI: 39.2%, 47.8%). This finding is consistent with a study conducted at the University of Gondar tertiary eye care and training (42.2%) and a university hospital (41.4%) (13, 22). The finding of the current study was higher than the study conducted in northwest Ethiopia (18.9%) (16), Arbaminch General Hospital, 13% (15), and systematic review and meta-analysis in Ethiopia (19.48%) (10), and in India (16.9%) (23). Different approaches, settings, DR risk comorbidities, diagnostic techniques, care quality, study participants, and health-seeking behaviors could all contribute to this variation in results between studies. However, the finding of the current study was lower than the study conducted in Sudan (82.6%) (24) and previous study on prevalence and impact on quality of life (75%) (25). These variations could be explained by variations in the research environment, genetics, diagnosis methods, assessment instruments, cultural background, and health-seeking behaviors. Furthermore, it could be the result of variations in the healthcare system and the standard of treatment provided to people with diabetes. In addition, the variation may be due to the current study conducted in an urban area where the patients may have better awareness about the complications of diabetic mellitus, including diabetic retinopathy.

The prevalence of DR among people with type 1 and type 2 diabetes was the main finding of the current investigation. The chi-square test revealed a statistically significant difference (P = 0.021) in the percentage of patients with type 1 and type 2 diabetes who develop diabetic retinopathy. The results demonstrated that the prevalence of diabetic retinopathy (DR) was found in individuals with type 1 diabetes (38.5%) and type 2 diabetes (48.5%). This study confirms that there is a more frequent eye complication in the 2DM group of patients. The prevalence of DR among type 1DM was higher and lower for type 2DM in a previous study conducted in northwestern Ethiopia (46.2%) and (41.7%) (26). This discrepancy may be due to the size of the study sample, the study conditions, and the availability and accessibility of medical care. Since the current study was conducted in urban countries, patients have access to different health services to early prevent the complications of diabetic mellitus. The other reason may be that the majority of the participants in the current study had a family history of diabetic mellitus, which may contribute to the higher prevalence of type 1 DM among the study participants. However, the findings of this study were in line with a comparable study carried out in northeast India, where the prevalence of DR among type 1 DM is 36.2% but is lower among type 2 DM (29.5%) (27). Another study carried out in north-east Poland showed that the prevalence of DR among type 1 DM was consistent (32.58%), but the prevalence of DR of type 2 DM for this study was higher (23.04%) (28).

In the current study, for both forms of diabetes, the following risk variables were identified: age, sex, glycemic status, comorbidity, and family history of diabetes.

Poor glycemic control is a significant risk factor for the development and progression of diabetic retinopathy (DR) in patients with diabetes mellitus (DM). Studies have consistently shown that individuals with poorly controlled blood glucose levels are at increased risk of developing DR compared to those with good glycemic control. This result is consistent with the study conducted in systematic reviews in China (29) Iran (30), Tanzania (31), and in Ethiopia Jimma University Hospital (22) in Southwest Ethiopia (32). Poor glycemic control can lead to abnormal glucose metabolism, activation of pathways that contribute to vascular damage, and increased production of reactive oxygen species, all of which can contribute to the development of diabetic retinopathy. Therefore, optimizing glycemic control through appropriate management strategies is essential to reduce the risk of DR in patients with diabetes (33).

The complications of diabetes mellitus (DM) play a crucial role in the development and progression of diabetic retinopathy (DR). Patients with complications of DM, such as hypertension, dyslipidemia, nephropathy, neuropathy, and peripheral vascular disease, have been found to have a significantly higher risk of developing DR compared to those without complications. This result was consistent with a study conducted in China (34), Tanzania (31), Kenya (35), Sudan (24), Arbaminch (15), and Jimma (22). Studies have consistently shown that the presence of complications from DM is strongly associated with an increased probability of developing diabetic retinopathy. Therefore, the management of DM complications is essential to prevent or delay the onset and progression of DR in people with diabetes. But this finding is not in line with a research project carried out in Iran (30). The technique, the confounding effects, the differences in self-care behaviors, and the differences in the prevalence of complications between studies could all be contributing factors to this variance.

Age is a crucial factor in the development and treatment of diabetes mellitus (DM) and its complications, including diabetic retinopathy (DR). Studies have shown that older age is associated with an increased risk of developing DR in people with type 1 and type 2 DM. Patients older than 60 years have been found to have a higher likelihood of developing DR compared to younger age groups. This result was corroborated by the study conducted in China (36), Tikur Anbessa Hospital, Ethiopia (37), and Southwest Ethiopia (32). This association highlights the importance of age as a risk factor for diabetic retinopathy and emphasizes the need for age-specific screening and management protocols in diabetic patients to prevent and detect DR at an early stage.

Male patients with diabetes mellitus (DM) have been shown to have a higher risk of developing diabetic retinopathy (DR) compared to female patients. Studies have reported that male DM patients are more likely to develop DR than their female counterparts. This evidence is supported by a study conducted in China (36) and the United Kingdom (38). This gender difference in the prevalence of DR among DM patients underscores the importance of considering gender-specific risk factors and customized interventions for the prevention and treatment of diabetic retinopathy in men with diabetes.

The family history of diabetes mellitus (DM) is a significant risk factor for the development of diabetic retinopathy (DR). Individuals with a family history of DM have a higher likelihood of developing DR compared to those without a history of this type. This result is in line with the study conducted in India (39) and Ethiopia (15, 32). This association underscores the genetic predisposition and the familial clustering of diabetes-related complications, including DR. Therefore, understanding the impact of the family history of DM on the risk of DR is crucial for early detection, prevention, and management strategies in people with a familial predisposition to diabetes.

The study calculated the sample size based on prevalence rates and statistical power, ensuring an adequate number of participants to draw meaningful conclusions. Some potential limitations of the study include: there may have been a selection bias in the study sample. As the participants were recruited from a single hospital setting, this could limit the generalizability of the findings to a broader population of diabetic patients. Since the study had a retrospective design, there could be limitations in data collection, medical record accuracy, and potential confounding variables that were not considered in the analysis. The study also did not control the influence of socioeconomic status and access to healthcare services on both diabetes management and DR development.

## Conclusion

In conclusion, the study conducted at Jugal Hospital provided valuable information on the prevalence of diabetic retinopathy (DR) among diabetic patients. The findings revealed a significant prevalence rate (43.5%) of diabetic retinopathy (DR) among the study population; the prevalence of diabetic retinopathy among type 1 DM (38.5%) was lower than the prevalence of DR among type 2 DM (48.5%).

The study identified several key risk factors associated with the development of DR, including poor glycemic control, older age, male sex, family history of diabetes mellitus, and complications related to diabetes. It requires implementing regular and systematic screening programs for diabetic patients to detect DR at an early stage, especially for those with poor glycemic control, older age, male sex, family history of DM, and diabetes-related complications.

## Data Availability

The raw data supporting the conclusions of this article will be made available by the authors, without undue reservation.
